# Flu Vaccinations in Pharmacies—A Review of Pharmacists Fighting Pandemics and Infectious Diseases

**DOI:** 10.3390/ijerph17217945

**Published:** 2020-10-29

**Authors:** Marcin Czech, Marcin Balcerzak, Adam Antczak, Michał Byliniak, Elżbieta Piotrowska-Rutkowska, Mariola Drozd, Grzegorz Juszczyk, Urszula Religioni, Regis Vaillancourt, Piotr Merks

**Affiliations:** 1 Department of Pharmacoeconomics, Institute of Mother and Child, 01-211 Warsaw, Poland; marcin.czech@biznes.edu.pl; 2 Medink eu, 05-500 Warsaw, Poland; marcin.balcerzak@medink.eu; 3 Polish Flu Vaccination Coalition, 00-061 Warsaw, Poland; adam.antczak@umed.lodz.pl; 4 Polish Pharmaceutical Chamber, 00-238 Warsaw, Polska; michal@byliniak.com (M.B.); prezes.nra@nia.org.pl (E.P.-R.); 5 Didactic Center, Department of Ethics and Medical Law, Department of Social Medicine, Inter-Faculty, Medical University of Lublin, 20-081 Lublin, Poland; md5058143@tlen.pl; 6 Department of Public Health, Medical University of Warsaw, 02-097 Warsaw, Poland; grzegorz.juszczyk@wum.edu.pl; 7 National Institute of Public Health—National Institute of Hygiene, 00-791 Warsaw, Poland; 8 Collegium of Business Administration, Warsaw School of Economics, 02-513 Warsaw, Poland; urszula.religioni@gmail.com; 9 Pharmacie Children’s Hospital of Eastern Ontario, Centre Hospitalier Pour Enfants de l’est de l’Ontario, 401 Smyth Road, Ottawa, ON K1H 8L1, Canada; rvaillancourt@cheo.on.ca; 10 Faculty of Medicine, Collegium Medicum, Cardinal Stefan Wyszyński University, 01-815 Warsaw, Poland; 11 Department of Pharmaceutical Technology, Faculty of Pharmacy, Collegium Medicum in Bydgoszcz, 85-067 Bydgoszcz, Poland; 12 Trade Union of Pharmacy Workers, 01-315 Warsaw, Poland; 13 Employed Pharmacist in Europe (EPhEU) Verband Angestellter Apotheker Österreichs (VAAÖ) Berufliche Interessenvertretung Spitalgasse 31/4, 1090 Vienna, Austria; 14 Polish Pharmaceutical Group, 91-342 Łódź, Poland

**Keywords:** vaccination, pharmacy, pharmacist, flu, COVID-19

## Abstract

The phenomenon of population ageing observed over recent years involves growing healthcare needs and the limited staffing and financing of healthcare systems, and as such demands some functional changes in the healthcare model in many countries. This situation is particularly significant in the face of a pandemic, e.g., flu, and currently COVID-19.As well as social education, preventive vaccinations are the most effective method of fighting the infectious diseases posing a special threat to seniors. Despite this, the vaccination coverage level in most European countries is relatively low. This is largely due to patients having limited access to vaccinations. In some countries, implementing vaccinations in pharmacies and by authorized pharmacists has significantly improved vaccination coverage rates and herd immunity, while lowering the cost of treating infections and the resulting complications, as well as minimizing the phenomenon of inappropriate antibiotic therapies. This article presents the role of pharmacists in the prevention of infectious diseases, pointing out the measurable effects of engaging pharmacists in conducting preventive vaccinations, as well as analyzing the models of implementing and conducting vaccinations in pharmacies in selected countries, and depicting recommendations regarding vaccinations developed by international organizations. The presented data is used to suggest requirements for the implementation of preventive vaccinations in community pharmacies.

## 1. Introduction

The outbreak of the COVID-19 pandemic in 2020 has been a challenge for healthcare systems all over the world [1,2], showing how crucial it is to develop effective methods of fighting infectious diseases. The determinants of achieving the expected results of these methods involve the demographic situation of a given country, medical resources, expenditures, and model of healthcare system. Cooperation between the various stakeholders of the healthcare system, such as medical communities, government representatives, and the media, is also of great significance. This issue is particularly important in the light of the population ageing observed in many countries [3,4], and also shortages of medical staff [5,6]. 

Meeting the challenges posed on healthcare systems, largely associated nowadays with the necessity of effective fighting infectious diseases, requires the engagement of many groups of medical staff. Here, the role of pharmacists should be emphasized. Despite being an essential element of the public healthcare system, their participation in efforts to prevent diseases, lengthen life, and promote health is relatively low. Apart from providing basic and advanced pharmaceutical services consisting in ensuring and optimizing pharmacotherapy, in the current situation, extended services focused on prevention, e.g., preventive vaccinations, such as flu vaccinations, are also essential [7]. 

In some European countries, preventive vaccinations are administered by pharmacists or another medical staff in community pharmacies [8], with the positive outcomes of these actions encouraging other countries to enable pharmacists to conduct infectious disease vaccinations [9,10]. This is particularly significant not only during the COVID-19 pandemic, but also due to large shortage in medical staff, the rapid population ageing, and the relatively low vaccination coverage percentage in high-risk patients [11,12].

The objective of this article is to aggregate existing knowledge in the effectiveness of engaging pharmacists to conduct preventive vaccinations, and to prove the role of pharmacists and pharmaceutical care in infectious disease prevention. Scientific literature review was carried out to summarize key aspects related to the vaccination provided by pharmacies and the role of the pharmacists in that process. Following searching strategy was applied: articles were extracted from PubMed using the terms “vaccine”, “vaccination” “vaccination coverage” “infectious diseases, “ and “vaccination implementation” in combination with “community pharmacy” and “community pharmacist”.

This article depicts the guidelines of international organizations and experiences connected with implementing models of flu vaccinations in pharmacies all over the world. Presentation of the current practices and outcomes of extending pharmaceutical services in these countries can foster discussion on the model of pharmacist involvement in conducting preventive vaccinations, especially against flu. 

## 2. Pharmaceutical Care in Preventing Infectious Diseases

Pharmaceutical service can be defined as the activities of pharmacists in the healthcare system to obtain a measurable benefit. Apart from providing access to high-quality medicinal products and the subsequent rational management, pharmaceutical services also include preventive medicine [13]. The provision of pharmaceutical services improves healthcare results and the quality of life of patients, and integrating these services with other healthcare services enables optimum use of the potential of pharmacies and pharmacists [14]. These activities support the healthcare system not only in disease prevention, but also in primary and specialist patient care (Table 1).

Infectious diseases among seniors are often severe and more likely to involve complications [15]. Preventive vaccinations are the most effective way to prevent various infections and the resulting complications, and thus bring a measurable benefit in the form of reduced costs of treatment of possible complications and subsequent prolonged hospitalization [16].

Despite the fact that a vaccination against COVID-19 has not been available so far, intense research is being conducted to develop one. The launch of a vaccine against COVID-19 will require the considerable involvement of medical staff to achieve a high vaccination coverage level [17]. A great deal of scientific evidence [18,19,20] indicates that authorizing pharmacists to administer vaccinations would significantly increase the number of vaccinated people in society. Although there is no registered vaccination against COVID-19, the American Society for Health-System Pharmacists has already issued recommendations to extend the authority of pharmacists to administer this vaccination in order to reduce the strain on doctors and nurses [21].

## 3. Vaccinations as the Main Method of Flue Prevention

Flu places a substantial burden not only on healthcare systems, but also on the entire economy [16,22]. Due to the possible severe complications (e.g., pneumonia, otitis media, and myocarditis), flu is particularly dangerous for children, people with chronic diseases, and those over 65 years old. The main method of specific primary flu prevention is by vaccination [17].

In December 2009, the Council of the European Union recommended attaining a flu vaccination coverage of 75% among seniors, and, if possible, to extend that to other risk groups [23]. The flu vaccination coverage levels differ significantly across European countries and do not reach the expected 75%. The highest vaccination rates are observed in Scotland, England, and Wales (72.8%, 70.5%, and 66.6%, respectively), the Netherlands (64.1%), Ireland (62.0%), and Portugal (60%). The lowest flu vaccination coverage rates are reported in Estonia (2%), Latvia (4.3%), and Poland (6.9%) [11].

Using all contact with patients by medical professions to actively promote vaccinations can greatly increase vaccination coverage. For this to work in principal, it is crucial to provide a broad access to vaccinations for all patients, and the vaccinations should be widely offered and administered in healthcare facilities, including community pharmacies. Although pharmacists constitute a small percentage of healthcare workers, patient access to pharmacies and their services, with no necessity to make appointments, longer opening hours and the convenient locations of pharmacies, enable reaching a wider group of patients, largely eliminating the coverage barriers to vaccinations [24]. The engagement of pharmacists in conducting preventive vaccinations is beneficial not only for patients, but also for doctors, nurses, and the entire healthcare system (Table 2).

A systematic review of studies on the effectiveness of vaccinations administered by pharmacists indicates that the vaccination coverage rates in these models are higher than in traditional systems of vaccinations [25]. Nevertheless, attention should be paid to the limited assessment of the effect of offering vaccinations by pharmacists in pharmacies on vaccination coverage among patients aged >65 years after those vaccinations were implemented, and where the vaccination coverage rates among people aged ≥65 years were usually high—several dozen percent. Estonia is the only country with a vaccination coverage rate of a few percent—the possibility of administering vaccinations in pharmacies was implemented in the 2019/2020 season. US data shows that people aged 65 and older living in the United States, where pharmacists have been authorized to administer vaccinations since 1997, had a significantly lower flu vaccination coverage rate than in people at the same age living in states where pharmacists were not authorized to administer vaccinations (now pharmacists can administer flu vaccinations in the whole United States) [20]. 

A similar situation was observed in Norway. Before implementing vaccinations in pharmacies, the flu vaccination coverage rate in Norway among people aged ≥65 years was relatively low (26.9% in the 2016/2017 flu season) [11]. A gradual implementation of vaccinations in pharmacies doubled the vaccination coverage rate in that age group [26] to 48% in the 2018/2019 season [27] and 59% in the 2019/2020 season [26].

## 4. Models of Implementing Vaccinations in European Pharmacies

**Figure 1:**
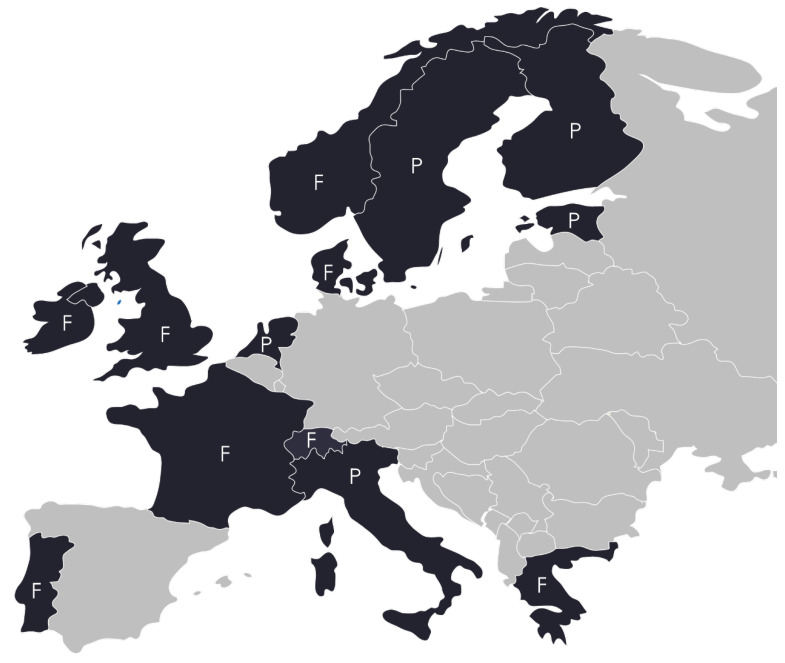
European countries with flu vaccinations available in pharmacies. F—the possibility of administering vaccinations in pharmacies by pharmacists; P—the possibility of administering vaccinations in pharmacies by other qualified healthcare professionals. Source: Antczak A, Balcerzak M, Byliniak M, Czech M, Drozd M, Merks P. Szczepienia przeciw grypie w aptekach. Raport opieka farmaceutyczna. Fundacja Nadzieja dla Zdrowia, Warszawa 2020.

There are a lot of models of implementing vaccinations in European pharmacies. In 2019, flu vaccinations were offered in pharmacies in 40% of European countries, and in 17%, other vaccinations were also available (*n* = 30) [28]. Currently, in Europe, vaccinations are administered in pharmacies in 13 countries (Figure 1), yet it should be noted that conducting vaccinations by pharmacies is not obligatory in any country. For example, in Great Britain and Portugal, vaccinations are available in about 78% of the pharmacies, and in Norway, in 60%. In some countries, the Czech Republic, Belgium, Germany, and others, endeavors are being made to be able to administer vaccinations in pharmacies, but they face opposition from other medical professions conducting vaccinations [9,10,29]. Due to the fact that vaccinations are dispensed and administered on the basis of prescriptions, most vaccination models are based on doctor-pharmacist cooperation. Pharmacists are authorized to administer vaccinations in Portugal, Ireland, Great Britain, France, Switzerland, Denmark, and Greece. In many countries, vaccinations are not conducted by pharmacists but by nursing staff employed for this purpose—this is the case in Italy, the Netherlands, Sweden, Finland, and Estonia. In some countries, e.g., in Portugal, there is a mixed model.

Selected models of implementing vaccinations in pharmacies, with the most advanced vaccination pattern in community pharmacies, where the health care model is similar to Poland’s health care system. 

### 4.1. Portugal

Portugal implemented the possibility of vaccination in the 2008/2009 flu season, being one of the first European countries in this respect. This was preceded by an information campaign. In the first flu season, 36.4% of all vaccinations were administered in Portuguese pharmacies. In the 2011/2012 flu season, nearly half of the vaccinations were conducted in pharmacies, although in the following years, due to changes in flu vaccination reimbursements that guaranteed people over 65 years of age free vaccinations in public healthcare facilities, the total number of vaccinations administered in pharmacies dropped by 25% in the 2014/2015 flu season [30].

The Portuguese Pharmaceutical Association developed guidelines on the implementation of services connected with administering vaccinations and medications in the form of injections in pharmacies, including minimum requirements for the provision of services and describing the process of basic and additional training sessions for pharmacists [31]. Having completed a basic training, Portuguese pharmacists are obliged to maintain the continuity of services and take part in a refresher training every five years. The guidelines regarding rooms, their equipment and vaccination reports were also developed. Currently, vaccinations are administered in 78% of all the pharmacies in Portugal [32].

### 4.2. Great Britain

In Great Britain, flu vaccinations could be administered by pharmacists in pharmacies from 2002, but only on a private basis. The problem of vaccination coverage led to engaging local pharmacies in 2005, where people over 65 years of age and younger people with risk factors are vaccinated. Implementing vaccinations in pharmacies was considered the main factor of the significant increase in the vaccination coverage level in 2008 [17]. From 2013, following development of the professional competence model and the procedures of patient qualification by the National Institute for Health and Care Excellence (NICE) and the Centre for Pharmacy Postsecondary Education (CPPE), contracts for services could be concluded between pharmacists and local NHS officials, determining the scope of the service, quality and responsibility for the service provided to patients. Since 2015, NHS has been contracting flu vaccinations as a nationwide service on the basis of annual National Flu Immunization Programs [33]. Currently, Great Britain is the European country with the highest vaccination coverage rate at nearly 75%. The implementation of vaccinations in pharmacies has significantly reduced the workload of primary healthcare [34].

### 4.3. France 

In 2017, a pilot project to increase the vaccination coverage rate was launched in France. It included nearly 3 thousand pharmacies located in four regions of France. A requirement for participation in the programme was the completion of special training sessions by pharmacists organized by the French Pharmaceutical Society (the project included over 5000 pharmacists). During the six months of the pilot project (September 2017–March 2018), 159,139 people were vaccinated against flu [35], which resulted in implementing vaccinations in pharmacies in the whole of France.

Currently, French pharmacists are authorized to administer flu vaccinations, and the law determines the staff qualifications and technical requirements for pharmacies. Pharmacists can administer flu vaccinations to adults, excluding patients with a history of allergic reactions. Patients can buy vaccinations in the pharmacy or receive it if they are entitled to free of charge vaccinations, and decide where the vaccination should be conducted. 

### 4.4. Norway 

Vaccinations in Norwegian pharmacies became an element of the national vaccination programme in 2018, preceded by a pilot project with 23 facilities [36]. Since that time, every year the number of pharmacies conducting vaccinations has increased (in the 2018/2019 flu season, vaccinations were available in 250 pharmacies; in the 2019/2020 season, in 600). In Norway, vaccinations in pharmacies are administered to people aged 12 years or older who have a prescription entitling to a vaccination. The objective of the implementation of vaccinations in Norwegian pharmacies was to increase the vaccination coverage rate and to limit the use of antibiotics by 30% in 2020, as a lower number of flu cases contributes largely to reducing the phenomenon of an inappropriate use of antibiotics [37]. 

## 5. Recommendations and Models of Implementing Flu Vaccinations in Pharmacies 

Conducting flu vaccinations in community pharmacies is recommended by numerous international organizations. The report “Global Influenza Strategy 2019–2030” prepared by the World Health Organization, encourages developing and implementing individual policies and programs optimized with respect to the national needs and to integrate them with the existing systems [38]. 

In this context, the European Observatory on Health Systems and Policies points out that limiting the possibility of administering flu vaccinations to just doctors is a great barrier in the execution of national vaccination programs. The availability of flu vaccinations in pharmacies is indicated as one of the effective initiatives in increasing flu vaccination rates [39]. 

In responding to the threat connected with infectious diseases and fears regarding vaccinations in April 2018, the Pharmaceutical Group of the European Union (PGEU) published a document presenting best practices within services provided by pharmacists throughout Europe. Vaccinations administered by pharmacists and another medical staff working in pharmacies included vaccinations against pneumococcus, herpes zoster, HPV, influenza, and vaccinations recommended for travelers [35].

The National Institute for Health and Care Excellence (NICE) [34] recommends a wide approach to the development and implementation of programs aimed at increasing flu vaccination rates. These programs should both raise awareness regarding vaccinations and offer them, and healthcare professionals responsible for vaccinations are obliged to acquire appropriate qualifications [40].

The guidelines for Good Pharmaceutical Practice by the International Pharmaceutical Federation (FIP and WHO) emphasize that good pharmaceutical practice (GPP) should entail education on vaccinations, encouraging patients to vaccinate, and administering vaccinations [41]. 

Centers for Disease Control and Prevention (CDC) highlight the key role of pharmacists in protecting patients against seasonal influenza and the resulting complications. The American Pharmacists Association (APhA) recommends that protective vaccinations be an all-year-round action. They developed detailed recommendations on implementing vaccinations in pharmacies [42], pointing out that immunization in pharmacies should commence with vaccinations against one disease and the scope of available vaccinations should be extended in the long run. According to APhA, influenza vaccinations are the best method to start immunization practices in pharmacies. They also indicate that pharmacy staff require training in the communication essential to educate and encourage vaccinations, and also in the safety of vaccinations. APhA recommends to document every vaccination, preferably by giving pharmacists access to existing systems of medical documentation. 

The American Society of Health-System Pharmacists (ASHP) recommends that pharmacists should fulfil a leading role in immunization screening [43]. In the latest guidelines from March 2020, ASHP calls for the development of the legal frameworks essential to involve pharmacists in actions at the healthcare system level aimed at fighting the COVID-19 pandemic, including vaccinating adults once a vaccination is available [17,21]. 

## 6. Requirements for Implementation of Vaccinations in Pharmacies—Recommendations

The implementation of vaccinations in community pharmacies requires some legal and organizational changes, as well as determining the way of financing vaccinations. To complete these tasks, the cooperation between government representatives, pharmacists, other medical professionals and patients is necessary, and the effectiveness of this cooperation will influence the success of the implementation of the developed model and outcomes.

### 6.1. Legal Requirements

Conducting preventive vaccinations by pharmacists needs to be preceded by appropriate legislative changes, the scope of which will depend on the existing legal solutions in a given country. Legislative works should concern the following issues:

→ training and granting authorization to pharmacists and changes in the pharmacy curriculum,
→ qualifying patients to vaccinations administered by pharmacists,
→ enabling pharmacists to conduct vaccinations,
→ dispensing vaccinations without submitting prescriptions (which can have a significant effect on the availability of vaccinations),
→ functioning of pharmacies as medical facilities,
→ logistic and organizational requirements for pharmacies (formal, legal, procedural, register, venue, sanitary),
→ keeping records of vaccinations,
→ ensuring patient rights,
→ responsibility of pharmacies and pharmacists for the consequences of vaccinations,
→ informing about the possibility to conduct vaccinations in pharmacies,
→ financing vaccinations [17].

Conducting vaccinations by pharmacists should be preceded by a system of appropriate training sessions and confirmation of acquired qualifications. Additionally, extending the framework pharmacy curriculum by classes in conducting preventive vaccinations should be considered. Similarly to other countries [30], they would include both theoretical and practical aspects. 

### 6.2. Organizational Requirements

Before conducting vaccinations, pharmacies should meet specific sanitary, organizational, logistic, and staff requirements. Rooms should ensure patient privacy when providing the service. They should include a chair for a patient (capable of reclining to a horizontal position as an alternative to a couch or sofa), a fridge for the vaccines, working space for preparing vaccinations, containers for ordinary and medical waste, hand sanitizer products, compresses, and dressings [44]. Pharmacies should be equipped with so-called anaphylactic kits [17].

Additionally, the work in pharmacies should be organized in such a way that the staff authorized to administer vaccinations are present for most of the time of the pharmacy hours. Studies show that convenient access to vaccinations is the main reason behind increased vaccination coverage rates [20,25,45,46]. Thus, the model enabling pharmacists to conduct vaccinations should give patients the opportunity to receive vaccinations at any time. Signing up for vaccinations or the possibility to conduct vaccinations only during certain hours apply in the countries (e.g., Sweden and Finland) where, despite no possibility to conduct vaccinations in pharmacies, this service must not be provided directly by pharmacists but by additional employed authorized staff. 

### 6.3. Economic Requirements

One condition for effective realization of vaccinations in pharmacies is appropriate remuneration to pharmacists for each vaccination, which should encourage them to provide this kind of service and to further develop pharmaceutical care. In Europe there are various mechanisms of payments for conducting vaccinations. For example, in Great Britain there is a complete reimbursement of the costs of vaccinations within the framework of the National Flu Immunization Programme, while in Portugal, the costs of vaccinations are borne by patients. For this reason, the implementation of vaccinations in community pharmacies requires an assessment of the possibility of financing vaccinations by public payers, which would be entail the necessity to conclude contracts between pharmacies and payers. In this context, all immunization cost elements, including cost of the vaccine, cold chain assurance, or service costs, should be considered.

## 7. Conclusions

Preventive actions are the most effective way of fighting infectious diseases. Efficient preventive actions, including preventive vaccinations, require involvement and cooperation between numerous groups of medical workers, namely pharmacists, doctors and nurses. The issue regarding patient access to vaccinations is not without significance. 

In several European countries it is possible to conduct preventive vaccinations such as flu vaccinations in community pharmacies. In many of these countries, pharmacists are authorized to administer vaccinations. Numerous reports indicate that the use of the models engaging pharmacists in preventive actions has resulted in increased vaccination coverage rates and herd immunity, and thus has a significant benefit on public health. Additional objectives achieved by healthcare systems thanks to the possibility to conduct vaccinations in pharmacies are:

→ To increase the state of readiness for action in the face of the pandemic,
→ To lower the costs of treatment of infectious diseases and the subsequent complications,
→ To limit unjustified use of antibiotics [17].

Development of a model of conducting vaccinations in pharmacies by pharmacists requires appropriate legal and organizational frameworks, as well as determining the method of financing these services. The development of pharmacist competencies and effective cooperation between the various stakeholders of healthcare are also of key significance here. 

At this point, the barriers that may arise from patients should also be emphasized. Anti-vaccination movements can be of particular importance. Patients’ concerns about vaccination, such as pain or fear of side effects, may also be significant. The role of the pharmacist in addressing patients’ concerns can be crucial. Pharmacists, being more accessible healthcare professionals who can devote much more time to the patient at a more convenient time, can significantly reduce patients’ fears and provide broad health education. However, this area requires further research.

## Figures and Tables

**Table 1:** The scope of pharmaceutical services and pharmaceutical care supporting primary and specialist healthcare.

Pharmacist Role in Healthcare		
Support in medical care	Asthma	− Monitoring the disease and therapy − Teaching inhalation techniques − Promoting compliance − Campaigns to identify non-controlled patients
	Chronic obstructive pulmonary disease	
	Diabetes	− Monitoring the disease and therapy − Promoting compliance − Campaigns to identify non-controlled patients
	Dyslipidemia	
	Hypertension	
	Bleeding disorders	− Monitoring the disease and therapy − Counselling or therapeutic education − Monitoring clinical parameters
	Obesity	− Counselling or therapeutic education − Monitoring clinical parameters − Campaigns to identify obese patients
	Support in primary healthcare	− Early diagnosis − Pain treatment − Monitoring depression therapy − Repeat prescriptions
	Support in specialist healthcare	− Dose adjustment of anticoagulants − Community pharmacies dispensing medications dispensed only by hospital pharmacies − Supporting patients in transition from hospital to outpatient treatment − Directly observed tuberculosis therapy − Early HIV detection
Mother and child health	Pregnancy/Breastfeeding	− Counselling − Techniques of using care products for pregnant women and their babies − Pregnancy tests
	Children	− Counselling − Techniques of using care products for children
Comprehensive interventions	Prescribing or administering medications	− Medications (including injections) − First aid − Vaccinations
	Assistance in home/nursing home therapy	− Counselling, consultations, monitoring − Home delivery of medications
	Counselling	− Dermocosmetics/Medical devices − OTC medicines − Dietary supplements − Veterinary medications
	Sun protection	− Counselling and campaigns
	Prevention programs	− Syringe exchange − Quitting smoking − Vaccinations
	Related to medications	− Education and improvement of health competences − Interventions increasing treatment adherence − Multiple doses of medications − Medication reviews − Home medicine cabinet reviews − Identifying drug interactions − Supervision over pharmacovigilance − Drug disposal programs

Source: Felix J, Ferreira D, Afonso-Silva M, Gomes MV, Ferreira C, Vandewalle B, Marques S, Mota M, Costa S, Cary M, Teixeira I, Paulino E, Macedo B, Barbosa CM. Social and economic value of Portuguese community pharmacies in health care. BMC Health Serv Res 2017;17:606; Antczak A, Balcerzak M, Byliniak M, Czech M, Drozd M, Merks P. Szczepienia przeciw grypie w aptekach. Raport opieka farmaceutyczna. Fundacja Nadzieja dla Zdrowia, Warszawa, 2020.

**Table 2:** Advantages of administering vaccinations in pharmacies for patients, medical staff and the healthcare system.

	Advantages of Administering Vaccinations in Pharmacies
Patients	− Removing a lot of organizational barriers to flu vaccinations − Increased availability of vaccinations (convenient places, longer opening hours of pharmacies) − Education of patients in the field of vaccinations (the possibility of reducing the patient’s concerns) − Decreased risk of flu and the resulting complications
Doctors	− Lower flu incidence − Reduced occupational burden − Decreased risk of secondary infections associated with a visit to a healthcare facility − Improved sanitary safety in medical facilities
Nurses	− Reduced occupational burden − Opportunity to cooperate with pharmacies about vaccinations
Pharmacists	− Increased responsibility for preventive actions within public healthcare − Professional development − Financial benefits
Healthcare system	− Increased vaccination coverage level and herd immunity − Using pharmacists’ competencies and availability for preventive actions in the healthcare system − Counteracting anti-vaccination groups by increased involvement of pharmacists in the promotion of vaccinations − Increased state of readiness to respond in the event of epidemic or pandemic − Lower costs of flu treatment (direct and indirect) − Decreased number of secondary infections of healthy people visiting healthcare facilities − Decreased unjustified use of antibiotics − Increased potential for giving first aid as a standard element of training of pharmacists administering flu vaccinations − Obtaining potential for changes in financing flu vaccinations

Source: Antczak A, Balcerzak M, Byliniak M, Czech M, Drozd M, Merks P. Szczepienia przeciw grypie w aptekach. Raport opieka farmaceutyczna. Fundacja Nadzieja dla Zdrowia, Warszawa 2020.
